# Integration of Breast Cancer Secretomes with Clinical Data Elucidates Potential Serum Markers for Disease Detection, Diagnosis, and Prognosis

**DOI:** 10.1371/journal.pone.0158296

**Published:** 2016-06-29

**Authors:** Yvonne S. Ziegler, James J. Moresco, John R. Yates, Ann M. Nardulli

**Affiliations:** 1 Department of Molecular and Integrative Physiology, University of Illinois at Urbana-Champaign, Urbana, Illinois, United States of America; 2 Department of Chemical Physiology, The Scripps Research Institute, La Jolla, California, United States of America; University of Alabama at Birmingham, UNITED STATES

## Abstract

Cancer cells secrete factors that influence adjacent cell behavior and can lead to enhanced proliferation and metastasis. To better understand the role of these factors in oncogenesis and disease progression, estrogen and progesterone receptor positive MCF-7 cells, triple negative breast cancer MDA-MB-231, DT22, and DT28 cells, and MCF-10A non-transformed mammary epithelial cells were grown in 3D cultures. A special emphasis was placed on triple negative breast cancer since these tumors are highly aggressive and no targeted treatments are currently available. The breast cancer cells secreted factors of variable potency that stimulated proliferation of the relatively quiescent MCF-10A cells. The conditioned medium from each cell line was subjected to mass spectrometry analysis and a variety of secreted proteins were identified including glycolytic enzymes, proteases, protease inhibitors, extracellular matrix proteins, and insulin-like growth factor binding proteins. An investigation of the secretome from each cell line yielded clues about strategies used for breast cancer proliferation and metastasis. Some of the proteins we identified may be useful in the development of a serum-based test for breast cancer detection, diagnosis, prognosis, and monitoring.

## Introduction

Breast cancer (BC) is the most commonly diagnosed cancer and the second leading cause of cancer-related deaths of women in the United States [1]. Nearly 230,000 women were diagnosed with BC and 40,000 died of this disease in the United States in 2015 [2]. The impact of this disease is not restricted to a single country but is a formidable worldwide health problem [3].

Although targeted treatments have been developed for tumors that express estrogen receptor α (ERα) and the progesterone receptor (PR) or overexpress the human epidermal growth factor receptor HER2, these tumors typically develop resistance to currently used treatments. Furthermore, triple negative breast cancer (TNBC) tumors, which fail to express ERα, PR, and HER2, have no approved targeted treatments. Thus, for relapsed tumors and for TNBC, the only treatments available are broad-spectrum chemotherapeutic drugs, which can result in debilitating and sometimes persistent side effects.

The poor prognosis for TNBC patients presents an especially acute problem for African American women. Although these women have a lower incidence of BC, African American women have a higher incidence of TNBC and a lower survival rate than their Caucasian American counterparts [4–6]. Furthermore, African American women are more likely to develop BC at an earlier age [7]. Women who are overweight, younger at initial diagnosis, from a lower socioeconomic group, or of Hispanic descent are also more likely to be diagnosed with TNBC [8]. To compound the problem, many of these women have more limited access to health care from prevention through diagnosis and treatment.

Mammography has been successful in the early detection of BC, but has also led to over-diagnosis [9] and resulted in aggressive treatment of tumors that may not have been destined to metastasize, at great medical and personal cost. The ability to detect BC with a serum-based test, also referred to as liquid biopsy, would significantly reduce the cost, inconvenience, and discomfort associated with mammography and would be a significant advancement. The adoption of newer technologies to detect even smaller tumors [10] could exacerbate the problem of over-diagnosis unless it is accompanied by additional information about tumorigenicity and aggressiveness. Thus, the ability to differentiate between aggressive and indolent tumors with a serum-based test could significantly impact the course of BC treatment. Indeed, some progress has been made in achieving a serum test for prostate cancer aggressiveness using a panel of 4 kallikrein proteins [11]. Ultimately, the synthesis of proteomic information with metabolomics [12] and genomics [13] could produce an exquisitely sensitive yet inexpensive test for BC diagnosis, treatment, prognosis, and monitoring.

With these challenges in mind, experiments were designed to identify proteins that are secreted by BC cells with a special emphasis on TNBC. Two well-characterized BC cell lines originally derived from pleural effusions were selected for our studies and included MCF-7 cells (ERα and PR positive) and MDA-MB-231 cells (TNBC). Importantly, both MCF-7and MDA-MB-231 cells have gene expression profiles that are similar to their respective tumor subtypes [14,15]. Two more recently isolated TNBC cell lines derived from primary tumors, DT22 and DT28 cells, were also included [16]. MCF-10A cells, which have been used extensively as a benign control, were chosen as a model of non-transformed mammary epithelial cells. Since cells grown on an extracellular matrix (ECM) more accurately reflect the *in vivo* context [17], three dimensional (3D) cultures were utilized. Conditioned medium (CM) was subjected to mass spectrometry (MS) analysis and the significance of selected proteins was examined using The Cancer Genome Atlas (TCGA) and Kaplan-Meier plots.

## Materials and Methods

### Cell lines and 2D cell cultures

MDA-MB-231, MCF-7, and MCF-10A cells were originally obtained from ATCC. Two TNBC cell lines recently derived from dissociated primary tumors (DT) were established as described [18] and were classified as basal claudin-low (DT22) and basal-epithelial (DT28) [16]. Cells were maintained as previously described [19].

### 3D cell cultures

3D cultures were prepared in 8 well chamber slides (Ibidi, Verona, Wisconsin) using an “on-top” protocol [17]. 50 μl Reduced Growth Factor Phenol Red Free Matrigel (BD Biosciences, Bedford, MA) was pipetted into each chamber of a chilled slide, carefully spread with the pipette tip to cover the entire well, and allowed to solidify at 37° for 30 minutes. In the interim, cells grown in 2D maintenance cultures were rinsed 2X with pre-warmed PBS, trypsinized, spun at 800 x g for 5 min., and resuspended in 200–400 μl of Assay Medium (DMEM/F12 with 2% FBS, 2% Matrigel, 5 ng/ml EGF, 0.5 μg/ml hydrocortisone, 0.1 μg/ml cholera toxin, 10 μg/ml insulin, and P/S). Cells were then diluted and counted in a hemocytometer and seeded at 10,000 cells/well. All 3D cultures were fed 3X weekly with 200 μl Assay Medium/well and were maintained at 37° in a 5% CO_2_ incubator for up to 21 days.

### Proliferation assay

3D cultures of the MCF-10A and BC cell lines were prepared in parallel. Media was removed from the MCF-10A cells, discarded, and replaced with 150 μl CM from other MCF-10A or BC cell cultures plus an additional 50 μl of fresh Assay Medium every 2–3 days for 14 days. CM was centrifuged at 1000 x *g* for 5 min. to remove any cells in suspension before adding back to the MCF-10A cell cultures. All cultures were followed by phase contrast microscopy for 14 days, pictures were taken with an Olympus XC30 camera mounted on an Olympus CKX41 phase contrast microscope, and cultures were fixed and stained for immunofluorescent confocal microscopy.

### Immunofluorescent staining

Staining was done essentially as described [19,20]. Briefly, cells were washed once with PBS and fixed in 4% paraformaldehyde for 20 minutes at room temperature. The paraformaldehyde was quenched and washed out with three rinses of PBS with 100 mM glycine and the cells were permeabilized with 0.5% Triton-X 100 for 10 minutes at 4°C. The cells were incubated in primary block (PBS plus 0.05% Tween, 0.2% Triton-X-100, and 10% normal donkey serum, Jackson Immunoresearch, West Grove, PA) for 1–1.5 hour at room temperature, then in fresh primary block with 20 μg/ml Affinipure F(ab) fragment Donkey anti-mouse IgG (Jackson Immunoresearch) for an additional 30–40 minutes. After three washes with PBS, the cultures were treated with primary antibody diluted in PBS with 0.05% Tween 20 (Ki67, 18-0191Z, 1:100, Thermo-Fisher; Laminin V, MAB19562, 1:200, EMD Millipore) and incubated overnight at 4°C. Cells were rinsed three times using PBS with 0.1% Tween 20, treated with secondary antibody diluted in PBS with 0.05% Tween 20 (715-486-150 and 715-496-152, 1:200, Jackson Immunoresearch), and cultures were incubated for 1 hour at room temperature. Cells were rinsed three times with PBS with 0.1% Tween 20, nuclei were counterstained with DAPI for 15 minutes at room temperature, rinsed once with PBS, and fresh PBS was added to each culture well before imaging.

To determine the percent of proliferating MCF-10A cells, Image Pro Plus software (Media Cybernetics, Bethesda MD) was utilized to calculate the number of Ki67- and DAPI-stained nuclei in three fields, each containing 300–800 cells. Statistical significance was determined using a one-way ANOVA followed by Tukey HSD post-hoc test. P values less than 0.05 were considered significantly different from control values. All analyses were performed using Microsoft Excel.

### Confocal microscopy

Imaging was performed through the bottom of the chamber slides using a Leica Microsystems TCS SPE high resolution spectral confocal microscope and data were analyzed with Leica Application Suite, Advanced Fluorescence (LAS AF) software. Z-stacks were created in 2 micron intervals with 2 passes per xy plane to minimize background.

### Preparation of CM for MS analysis

MCF-10A and BC cells were grown in 3D cultures. CM was collected 3X a week from the 3D cultures. To ensure that any cells present in the media would be removed, the CM was centrifuged at 1000 x *g* for 5 minutes after collection. The CM from a total of 6 media changes was pooled and the pooled samples were subjected to MS analysis.

### Mass spectrometry

Pooled CM (100 μg protein) from 3D cultures was precipitated in 23% trichloroacetic acid. Acetone washed pellets were dissolved in 100 mM Tris pH 8.5 with 8 M urea. Proteins were reduced with 5 mM Tris(2-carboxyethyl)phosphine hydrochloride (Sigma-Aldrich, St. Louis, MO, product C4706) and alkylated with 10 mM iodoacetamide (Sigma-Aldrich, St. Louis, MO, product I11490). Proteins were digested for 18 hr at 37°C in 100 mM Tris pH 8.5 with 2 M urea, 1 mM CaCl_2_, and 2 ug trypsin (Promega, Madison, WI, product V5111). Digestion was terminated with 5% formic acid. Debris was removed by centrifugation for 30 min at 18000 x g. Unless otherwise noted all chemicals were purchased from Thermo Fisher Scientific (Waltham, MA). Deionized water (18.2 MΩ, Barnstead, Dubuque, IA) was used for all preparations.

A MudPIT microcolumn [21,22] was prepared as described [19] and LC-MS/MS analysis was performed using an Easy-nLC (Thermo) and an LTQ (Thermo) using an in-house built electrospray stage. 11-step MudPIT experiments were performed where each step corresponds to 0, 10, 20, 30, 40, 50, 60, 70, 80, 90 and 100% Buffer C (500 mM ammonium acetate, 5% acetonitrile 0.1% formic acid) being injected at the beginning of a 105 min gradient. Precursor scanning was performed from 300–2000 m/z. Data-dependent acquisition of MS/MS spectra were performed with the following settings: MS/MS on the 5 most intense ions per precursor scan. Dynamic exclusion settings used were as follows: repeat count, 1; repeat duration, 30 second; exclusion list size, 50; and exclusion duration, 180 seconds.

Protein and peptide identification and modified peptide analysis were done with Integrated Proteomics Pipeline—IP2 (Integrated Proteomics Applications, Inc., San Diego, CA. http://www.integratedproteomics.com/) using ProLuCID, DTASelect2. Spectrum raw files were extracted into ms2 files from raw files using RawExtract 1.9.9 (http://fields.scripps.edu/downloads.php) [23] and the tandem mass spectra were searched against a human protein database (UniProtKB- Swiss-Prot, reviewed) [24]. In order to accurately estimate peptide probabilities and false discovery rates, a decoy database containing the reversed sequences of all the proteins appended to the target database was utilized [25]. Tandem mass spectra were matched to sequences using the ProLuCID algorithm [26]. The search space included half and fully tryptic peptide candidates that fell within the mass tolerance window with no miscleavage constraint. Carbamidomethylation (+57.02146 Da) of cysteine was considered as a static modification. DTASelect parameters were -p 2 -y 1—pfp .01—extra—pI—DB—dm -in.

Following MS, a list of secreted proteins was generated (FDR p≤0.02). The list was then curated to remove proteins highly represented in the medium alone and peptide files were utilized to minimize redundant identifications. In addition to the SC data used throughout the manuscript, data were also calculated using a normalized spectral abundance factor (NSAF) which accounts for the effect of protein length on the number of SCs. For brevity, non-italicized gene names will be used to reference proteins and RNA transcripts throughout the paper.

### Western blotting

A portion of the CM prepared for MS was saved for Western blotting analysis. The protein concentration of each sample was determined using the bicinchoninic acid (BCA) assay (Thermo Scientific) with bovine serum albumin (BSA) as a standard. 25 μg protein was loaded onto each lane of a denaturing 4–12% gradient gel and fractionated. Proteins were transferred to a nitrocellulose membrane and the blots were probed with a CTSD- (sc10725, Santa Cruz Biotechnologies), ECM 1- (ab126629, Abcam), PRDX1- (ab109498, Abcam), or SFN- (ab14123, Abcam) specific antibody. The SFN sample was concentrated up to 6-fold using Amicon Ultra-0.5 Centrifugal Filter 3K Devices (Millipore). Western blots were imaged and quantitated with a Licor Odyssey Infrared Imaging System.

### TCGA data extraction and boxplots

Gene expression in human BC tumors is derived from the TCGA Research Network: http://cancergenome.nih.gov/. TCGA level 3 gene expression data (interpreted/normalized) for breast invasive carcinoma, tumor-matched normal, was downloaded and parsed with a script generated by Jeffrey Haas at the University of Illinois Life Sciences Office of Information Technology (S1 Table). Normal tissue, as defined by TCGA, represents pooled normal mammary tissue from a number of individuals that are not matched to the tumor donors. Data for luminal A (209 tumors) and TNBC (80 tumors) classifications are presented as boxplots to visualize average expression as well as variability in the expression within a clinical category.

Boxplots were created by importing TCGA data into Plotly, an on-line analytics and data visualization tool (URL: https://plot.ly).

### Kaplan-Meier recurrence-free survival curves

Curves were generated based on individual gene expression using a Kaplan-Meier plotter (kmplotter.com) for BC [27]. Data were analyzed using the JetSet best probe set, with no restrictions on subtypes or cohorts, and with quality control settings including (i) remove redundant samples, (ii) exclude biased arrays, and (iii) check proportional hazards assumption.

## Results and Discussion

**Fig 1:**
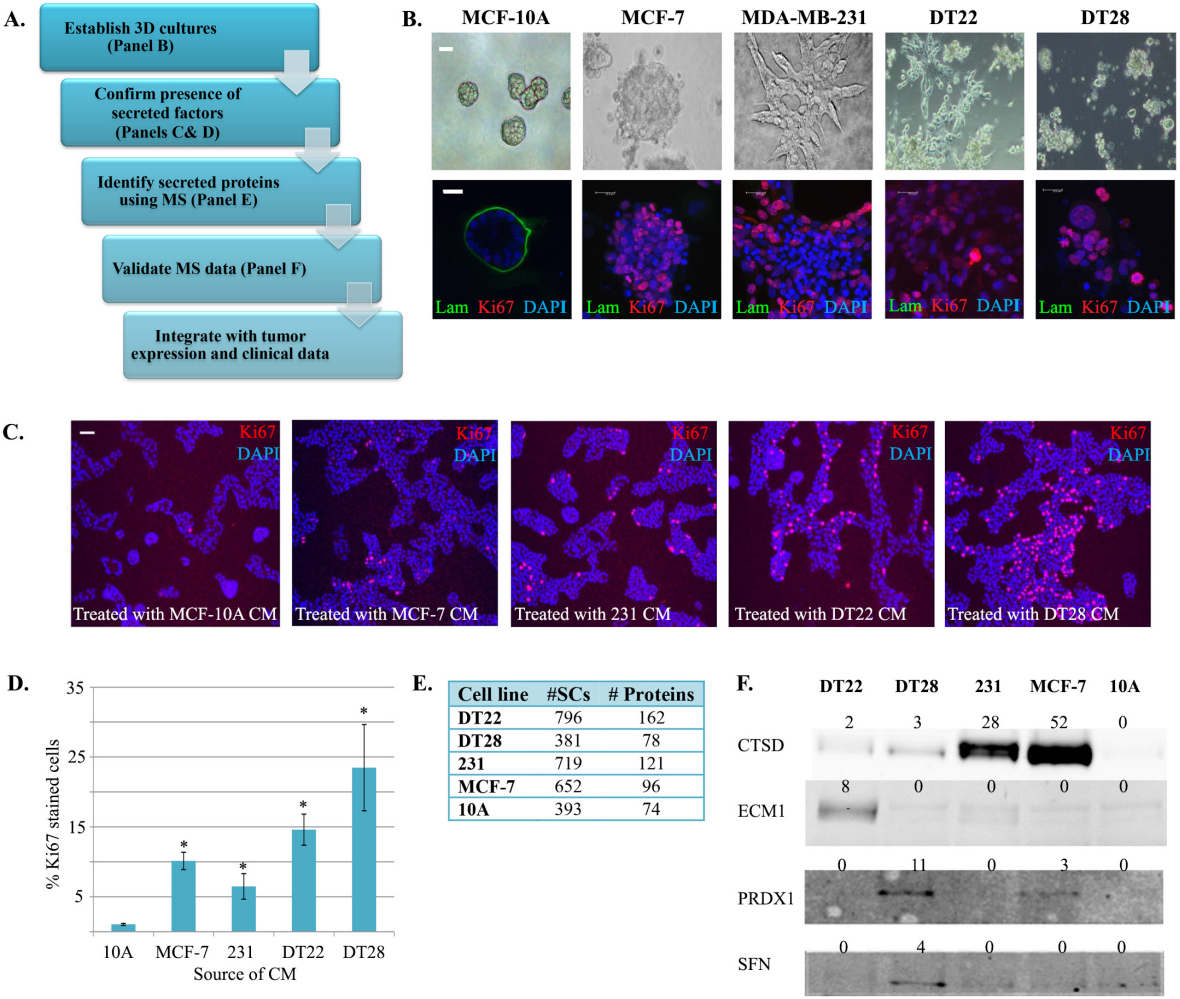
Identification of proteins in BC and MCF-10A cell secretomes. A. A workflow was established to analyze the secretomes of BC and benign mammary epithelial cells. B. Benign (MCF-10A), ERα-positive BC (MCF-7), and TNBC (MDA-MB-231, DT22, and DT28) cells were established in 3D cultures using Matrigel extracellular matrix. Live (top row) and fixed (bottom row) cultures were imaged by phase contrast or immunofluorescence, respectively. Markers of proliferation (Ki67-red), basement membrane (laminin-green), and nuclei (DAPI-blue) were utilized. C. 3D cultures of MCF-10A cells were exposed to CM from MCF-10A, MCF-7, MDA-MB-231, DT22, or DT28 cells. Proliferation (red) and nuclei (blue) are indicated by Ki67 and DAPI staining, respectively. Scale bars indicate 25 μm. D. 3D cultures described in panel C were analyzed to determine the percent of proliferating MCF-10A cells for each treatment. An asterisk (*) indicates a p-value <0.05. E. CM from each cell line was subjected to MS and compiled results are shown. F. CM from 3D cultures of BC and MCF-10A cells were subjected to Western blotting analysis using antibodies to cathepsin D (CTSD), extracellular matrix protein 1 (ECM1), peroxiredoxin 1 (PRDX1), or 14-3-3 sigma (SFN). The number of spectral counts (SCs) is indicated above each lane. To visualize SFN, the CM was concentrated before blotting. MDA-MB-231 and MCF-10A cells are labeled as 231 and 10A, respectively. (Abbreviations: BC = breast cancer, TNBC = triple negative breast cancer, 3D = three dimensional, CM = conditioned medium, MS = mass spectroscopy)

The overall goal of these experiments was to (i) define the secretomes of different types of BC cells, (ii) interrogate the data to compare secretory behavior of benign, non-metastatic, and highly metastatic cell lines, (iii) determine the relevance of selected identified proteins by analyzing tumor expression and recurrence-free survival, and (iv) ascertain whether any of the secreted proteins have been previously identified in the serum of cancer patients (Fig 1A). Rather than using BC cell lines that were grown on plastic as previously reported [28], 3D cultures of cells were grown on Matrigel that more closely recapitulates the mammary gland microenvironment than the 2D cultures typically employed [17,29,30]. Furthermore, instead of examining the effluent from minced primary tumors comprised of multiple cell types [31,32], our studies examined the secretomes of non-transformed and malignant mammary epithelial cells. In so doing we would be ensured that the proteins we identified were derived from the single population of cells most intimately involved in tumorigenesis, epithelial cells.

The first step of our project was to establish a 3D culture system. Human mammary epithelial cells were seeded into microslide chambers that had been coated with Matrigel. The Matrigel not only functioned as a substrate for the cells, but was also included in the media to more closely mimic the *in vivo* mammary microenvironment and promote cell organization [33].

### 3D culture morphologies

When 3D cultures of mammary epithelial cells were established, the MCF-10A cells formed similarly sized hollow spheres with discrete basement membranes (Fig 1B, green stain) reminiscent of acini present in the normal mammary gland. Once formed, the acini exhibited a very low proliferation rate as indicated by the lack of red Ki67 staining. In contrast, MCF-7 cells formed irregularly-sized and shaped masses with no basement membrane or distinct lumen. Ki67 staining demonstrated that these cells were highly proliferative. MDA-MB-231 cells formed stellate structures lacking a basement membrane and were highly proliferative. MCF-10A, MCF-7, and MDA-MB-231 cells all behaved in 3D culture as previously described [20,34]. However, 3D cultures of the 2 other TNBC BC cell lines (DT22 and DT28) have not been examined. The DT22 cells formed multicellular stellate structures with long cellular processes and DT28 cells formed irregularly-sized grape-like clusters. Neither DT22 nor DT28 cells formed a basement membrane and both were highly proliferative.

### Evidence for soluble secreted proteins in 3D cultures

BC cells secrete factors [35] and exosomes [36] into their environment that can cause surrounding normal cells to take on tumorigenic characteristics, induce proximal stromal and immune cells to become tumor supportive, and influence distal tissues to become attractive future metastatic sites [37]. To determine whether our 3D cultures secreted biologically active factors that influence benign cell function, MCF-10A cells were exposed to CM from 3D cultures of MCF-10A (negative control), MCF-7, MDA-MB-231, DT22, or DT28 cells. Importantly, significantly greater increases in proliferation were observed when MCF-10A cells were treated with CM from each of the BC cells than with CM from other MCF-10A cells (Fig 1C and 1D). These findings demonstrated that active factors were being secreted by all of the BC cell lines and that these factors significantly stimulated proliferation of the benign MCF-10A cells.

### Overview of MS results

Since CM from the BC cells stimulated the growth of MCF-10A cells, we used MS analysis to identify proteins in the CM from 3D cultures of DT22, DT28, MDA-MB-231, MCF-7, and MCF-10A cells (S2 Table). Since the number of spectral counts (SCs) is strongly correlated with protein abundance [38], SCs were used to compare protein secretion. A minimum of 2 SCs was required for inclusion in the data. For comparison, normalized spectral abundance factor (NSAF) data is provided in S2 and S3 Tables. There was a wide variability in the number of spectral counts (SCs) and the number of proteins secreted by the mammary epithelial cells. The highest secretors were two of the TNBC cell lines, DT22 (796 SCs, 162 proteins) and MDA-MB-231 (719 SCs, 121 proteins) cells (Fig 1E). Interestingly, the third TNBC secretome examined (DT28) yielded only 381 SCs and 78 proteins, which was similar to the number of SCs (393) and proteins (74) in the MCF-10A cell secretome. MCF-7 cells secreted a moderate number of SCs (652) and proteins (96).

Current MS technology can yield data that is semi-quantitative in nature [38,39]. To determine whether the MS data faithfully reflected the levels of protein present in the CM from each of the cell lines, CM was subjected to Western blotting analysis. In fact, there was a good correspondence between the number of SCs generated by MS analysis and the intensities of the cathepsin D (CTSD), extracellular matrix protein 1 (ECM1), peroxiredoxin 1 (PRDX1), and 14-3-3 sigma (SFN) bands on Western blots (Fig 1F). This was true even for SFN, which was present at such a low level that the CM had to be concentrated prior to Western blotting.

An overview of the secreted proteins identified by MS analysis revealed a natural partitioning into several functional groups including glycolytic enzymes, proteases, protease inhibitors, potential exosomal proteins, insulin-like growth factor binding proteins (IGFBPs), and ECM proteins. To compare the BC cell lines to breast tumors, The Cancer Genome Atlas (TCGA) BC database was used to analyze gene expression of luminal A tumors, modeled by MCF-7 cells, and TNBC tumors, modeled by MDA-MB-231, DT22, and DT28 cells. Although TCGA data are derived from heterogeneous tumors comprised of epithelial, stromal, vascular, and immune cells, and some proteins are present intracellularly as well as secreted, many of the comparisons were enlightening. In order to evaluate clinical significance, Kaplan-Meier recurrence-free survival curves were utilized to investigate how the expression of selected secreted proteins relates to disease-free survival of BC patients [27].

### Glycolytic enzymes

Cancer cells have long been known to utilize the glycolytic pathway even in the presence of adequate oxygen [40]. This phenomenon, known as the Warburg effect, is thought to be the result of damaged mitochondria, hypoxic stress, and/or the increased energy demands of transformed, proliferating cells. However, it is also known that many of the glycolytic enzymes are “moonlighters”, proteins with multiple functions [41,42], and that some of these proteins may play a role in carcinogenesis [43]. For example, GAPDH, in addition to its classic role in glycolysis, is involved in apoptosis, nitrosylation of nuclear proteins, regulation of mRNA stability, and iron transport [44]. Also, glucose-6-phosphate isomerase (GPI) has established roles as a cytokine and growth factor in extracellular processes including cell motility and invasion [45,46].

**Fig 2:**
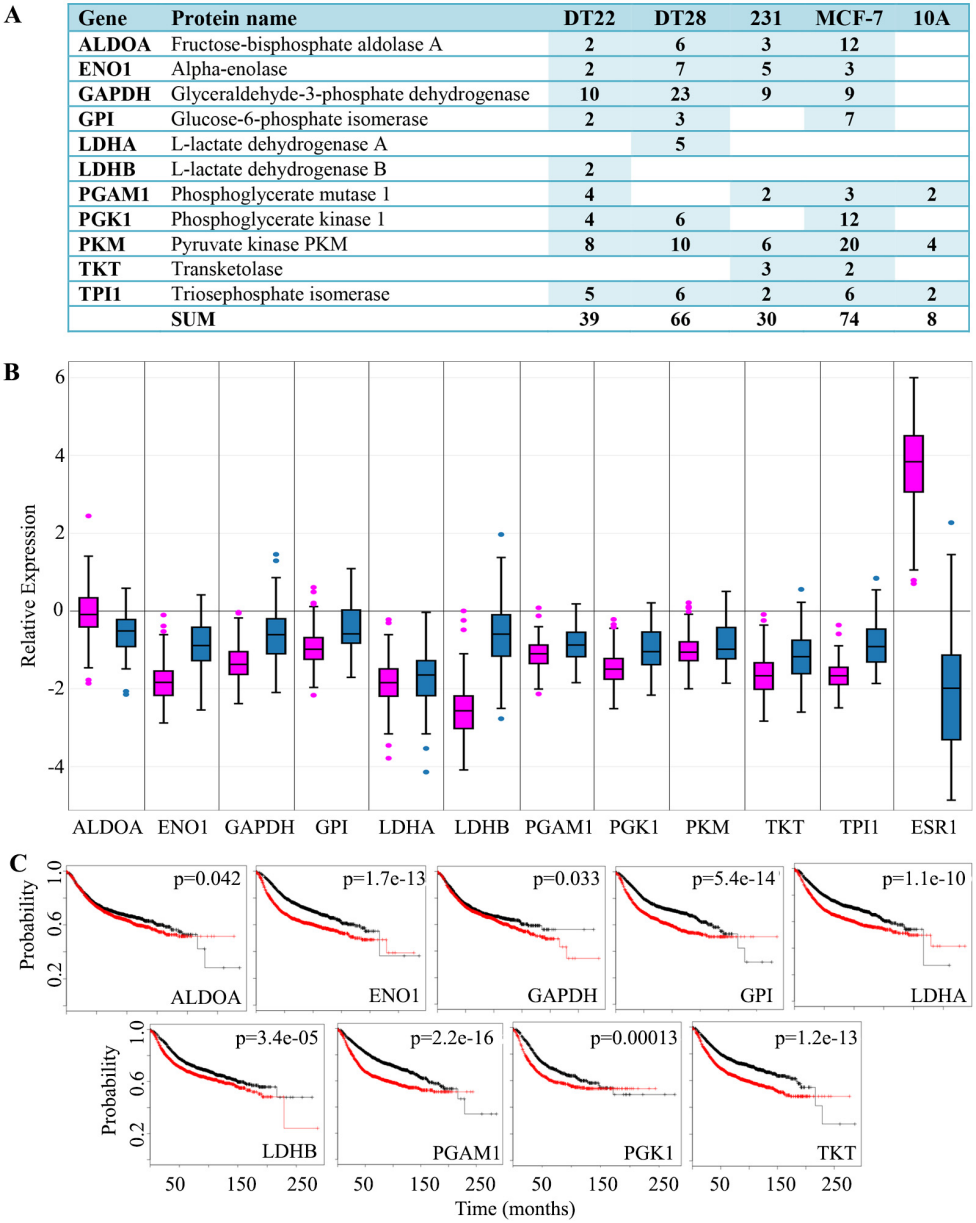
Glycolytic enzymes detected in the CM of BC and MCF-10A cells. A. The number of SCs identified in the CM of each cell line is indicated. A blank cell indicates that no SCs were detected. Both the HUGO gene symbol and Uniprot recommended protein name are provided. MDA-MB-231 and MCF-10A cells are labeled as 231 and 10A, respectively. NSAF data can be found in S3 Table. B. BC gene expression data was extracted from TCGA and boxplots were created for luminal A (pink) and TNBC (blue) tumors. ERα (ESR1) was included for comparison. Expression values are log2 normalized, tumor matched normal, with normal mammary tissue expression set to 0. C. Kaplan-Meier survival curves for human BC patients were created for 9 of the glycolytic enzymes secreted by BC cells in 3D cultures. The x axis represents the months of recurrence-free survival. Red and black curves indicate higher and lower expression, respectively. The p-value for each result is shown in the upper right quadrant. (Abbreviations: CM = conditioned medium, BC = breast cancer, SC = spectral counts, TCGA = The Cancer Genome Atlas, TNBC = triple negative breast cancer, 3D = three dimensional)

Numerous glycolytic enzymes were present in the secretomes of the cells we examined (Fig 2A). Interestingly, each of the BC cell lines secreted far more glycolytic proteins (30–74 SCs) than MCF-10A cells (8 SCs). MCF-7 cells, which are ERα-positive, were the highest secretors of these glycolytic enzymes, but the TNBC cell lines also secreted substantial amounts of these proteins. Interestingly, plasma samples from women with ERα-positive BC have elevated levels of several of these enzymes and the levels increase as the disease progresses [47]. Since each of these enzymes is also a highly expressed intracellular protein, one might anticipate that the TCGA expression data would not vary substantially compared to other well-known proteins that are up-regulated in BC such as ERα (ESR1) in luminal A tumors (Fig 2B). However, we were surprised that the average expression of the glycolytic genes is not increased in BC tumors, but is in fact decreased in BC tumors compared to normal mammary tissue. Nonetheless, TNBC tumors consistently exhibited higher expression of glycolytic genes compared to luminal A tumors with the exception of ALDOA. Intriguingly, when examining recurrence-free survival, higher expression of nine of these eleven glycolytic proteins (ALDOA, ENO1, GAPDH, GPI, LDHA, LDHB, PGAM1, PGK1, TKT) predicts a more rapid disease progression (Fig 2C) indicating the importance of these proteins in patient survival. The diagnostic and therapeutic potential of the secreted glycolytic proteins is a potentially rich field for further investigation.

### Proteases and protease inhibitors

The ECM provides the support for cells to form tissue, but as a tumor develops and initiates the process of metastasis, the ECM is modified. An overall deregulation of the protease-protease inhibitor balance in cancer has been well-documented [48] and many studies point to a correlation between the secretion of proteases and cancer progression [49,50].

**Fig 3:**
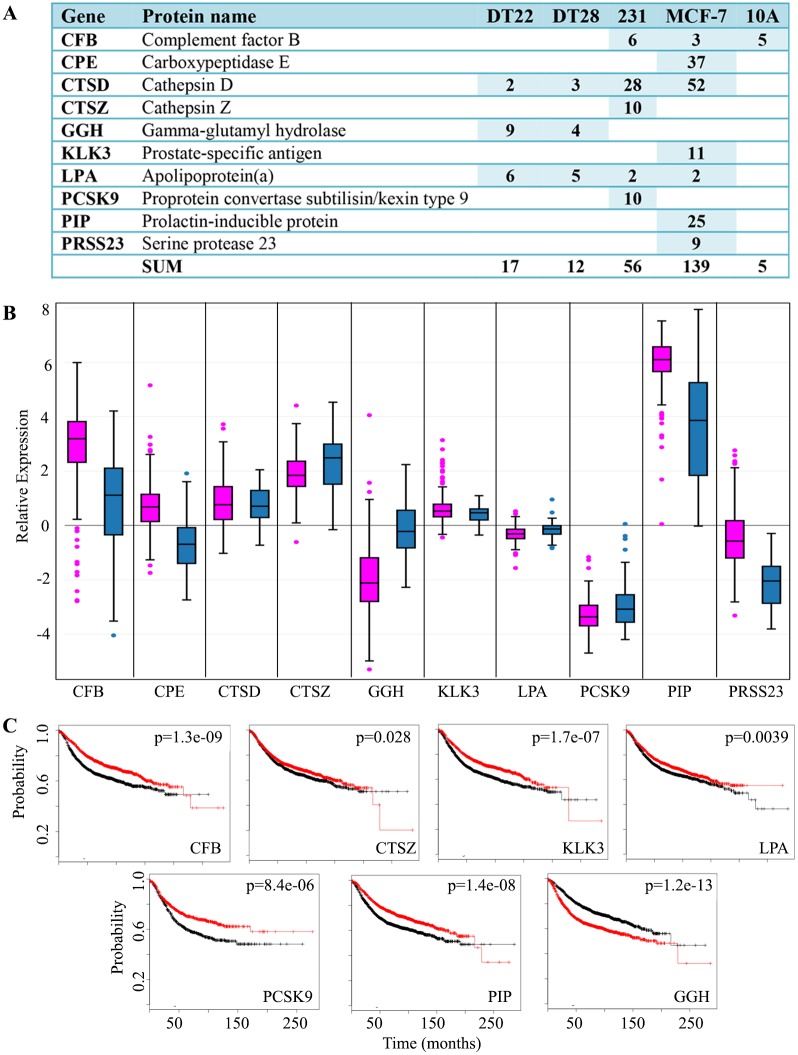
Proteases detected in the CM of BC and MCF-10A cells. A. The number of SCs identified in the CM of each cell line is indicated. MDA-MB-231 and MCF-10A cells are labeled as 231 and 10A, respectively. NSAF data can be found in S3 Table. B. BC gene expression data was extracted from TCGA and boxplots were created for luminal A (pink) and TNBC (blue) tumors. Expression values are log2 normalized, tumor matched normal, with normal mammary tissue expression set to 0. C. Kaplan-Meier survival curves for human BC patients were created. The x axis represents the months of recurrence-free survival. Red and black curves indicate higher and lower expression, respectively. The p-value for each result is shown in the upper right quadrant. (Abbreviations: CM = conditioned medium, BC = breast cancer, SC = spectral counts, TCGA = The Cancer Genome Atlas, TNBC = triple negative breast cancer)

The collection of proteases that act to modify the cellular microenvironment has been defined as the “degradome” [51]. A more complete understanding of the degradome could lead to therapeutics that target cancer growth and metastasis. Most pertinent to this study, BC patients shed significantly more peptide products of protease digestion into their plasma than healthy individuals [52], and this is in agreement with the many studies that have implicated the role of proteases in cancer progression [48]. A number of secreted proteins with protease activity were identified in the CM of the BC cells including serine, lysosomal, and aspartic proteases and metalloproteinases (Fig 3A). Intriguingly, the number of protease SCs detected in the CM of MCF-7 cells (139 SCs), which do not metastasize in mouse models, was far greater than the number of protease SCs detected in the CM of the highly metastatic MDA-MB-231 cells (56 SCs). Uniquely included in the secretome of MCF-7 cells were carboxypeptidase E (CPE), kallikrein-related peptidase 3 (KLK3), prolactin-inducible protein (PIP), and serine protease 23 (PRSS23). CPE, which cleaves C-terminal amino acids in the biosynthesis of a variety of critical peptide hormones and neurotransmitters, may also be involved in tumor growth and metastasis [53]. KLK3, more widely known as prostate-specific antigen or PSA, is a serine protease that is widely used clinically to detect prostate cancer and monitor its progression. PIP, an aspartyl protease that degrades fibronectin, has been implicated in BC proliferation and invasion [54]. The serine protease PRSS23 is up-regulated in ERα-positive BC as well as prostate, thyroid, and pancreatic cancer and has been associated with BC cell proliferation [55]. Cathepsin D (CTSD) was highly represented in the CM of MCF-7 (52 SCs) and MDA-MB-231 (28 SCs) and has been described as a proangiogenic factor in epithelial ovarian cancer [56] and an enhancer of BC metastases [57]. Cathepsin Z (CTSZ) was unique to the MDA-MB-231 secretome. CTSZ overexpression has been implicated in the epithelial to mesenchymal transition of liver cancer cells [58]. There were 17 or fewer protease SCs in the CM of DT22, DT28 and MCF-10A cells. DT22 cells have not been shown to be metastatic in a mouse model system [16]. The low number of proteases secreted by the metastatic DT28 cells [16] is intriguing and may reflect alternative strategies for migration.

Although the expression of proteases is highly variable in human mammary tumors (Fig 3B), the CM data corresponds well with tumor data in that genes that are more highly expressed in luminal A tumors are more highly or uniquely represented in the CM of MCF-7 cells (CPE, CTSD, KLK3, PIP, PRSS23), and genes more highly expressed by TNBC tumors are more highly or uniquely represented in the CM of DT22, DT28, or MDA-MB-231 cells (CTSZ, GGH, PCSK9).

The role of proteases in cancer progression and metastasis has been well documented [48]. Thus, it is perplexing that an examination of recurrence-free survival statistics reveals a slower progression of disease when 6 of these 10 protease genes are more highly expressed (Fig 3C, CFB, CTSZ, KLK3, LPA, PCSK9, PIP). Of the remaining proteases identified, the expression of 3 do not appear to influence disease progression (CPE, CTSD, PRSS23- data not shown) and only GGH is associated with more rapid disease progression. Interestingly, GGH was only detected in the CM of DT22 and DT28 cells, both of which were derived from primary TNBC tumors [16].

**Fig 4:**
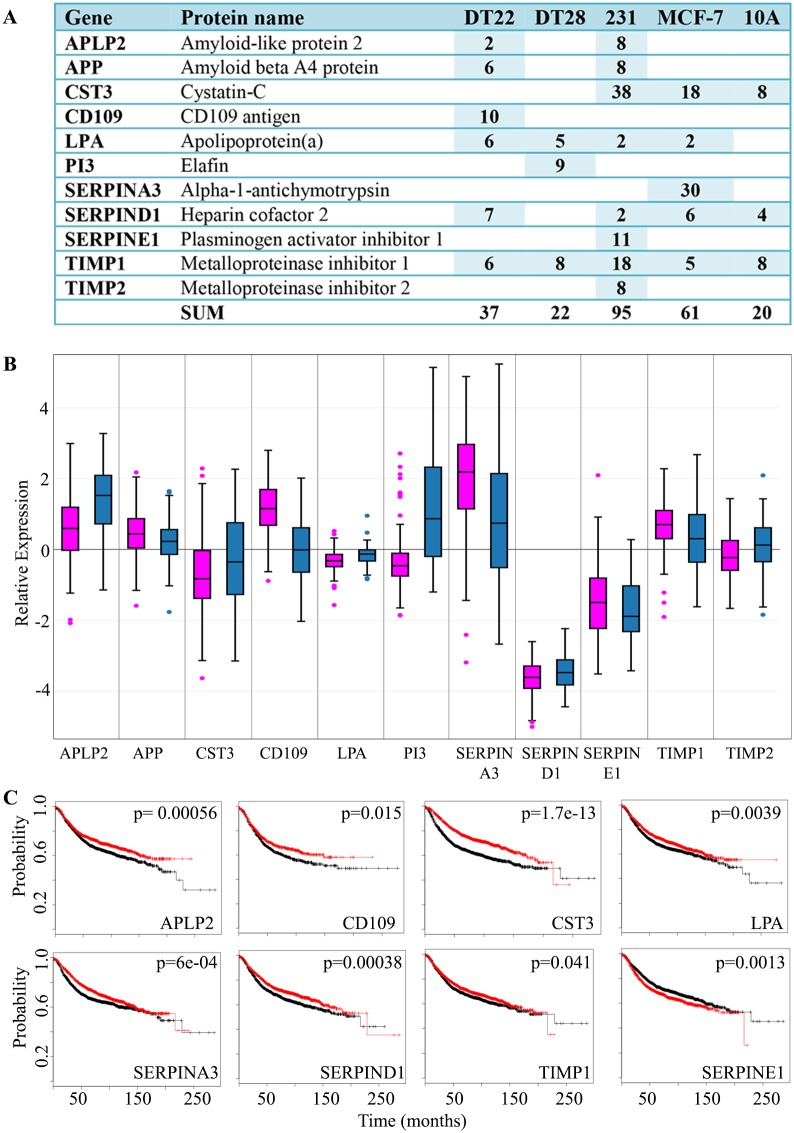
Protease inhibitors detected in the CM of BC and MCF-10A cells. A. The number of SCs identified in the CM of each cell line is indicated. MDA-MB-231 and MCF-10A cells are labeled as 231 and 10A, respectively. NSAF data can be found in S3 Table. B. BC gene expression data was extracted from TCGA and boxplots were created for luminal A (pink) and TNBC (blue) tumors. Expression values are log2 normalized, tumor matched normal, with normal mammary tissue expression set to 0. C. Kaplan-Meier survival curves for human BC patients were created. The x axis represents the months of recurrence-free survival. Red and black curves indicate higher and lower expression, respectively. The p-value for each result is shown in the upper right quadrant. (Abbreviations: CM = conditioned medium, BC = breast cancer, SC = spectral counts, TCGA = The Cancer Genome Atlas, TNBC = triple negative breast cancer)

Protease inhibitors were also well represented in our 3D cell secretomes (Fig 4A). Each of the cell lines secreted more protease inhibitors than proteases except for MCF-7 cells (Compare Fig 3A to Fig 4A). Serine protease inhibitors, cysteine protease inhibitors, and metalloproteinase inhibitors were present. Like the proteases, expression of protease inhibitors is highly variable in human mammary tumors (Fig 4B). Of the 11 protease inhibitors identified, higher expression of 7 of these inhibitors is associated with slower disease progression (APLP2, CD109, CST3, LPA, SERPINA3, SERPIND1, TIMP1), 3 did not influence disease progression (APP, PI3, TIMP2- data not shown), and only SERPINE1 is associated with more rapid disease progression (Fig 4C). Paradoxically, MCF-7 cells, which do not metastasize in mouse model systems, were the highest secretors of proteases (Fig 3A) and the highly metastatic MDA-MB-231 cells were the highest secretors of protease inhibitors (Fig 4A). It seems likely that, rather than a single protein mediating cell migration, the combined effects of multiple proteases and protease inhibitors influence the ability of BC cells to migrate.

### Prostate-specific antigen and breast cancer

Of particular interest was prostate-specific antigen (PSA, gene name KLK3), a multifunctional serine protease that has been used extensively for serum detection of prostate cancer. Interestingly, PSA was uniquely secreted by MCF-7 cells (Fig 3A). Although not thought of as a BC marker, PSA has been described in both male and female primary breast carcinoma [59]. Reports of PSA expression in BC are limited and correlated with ERα, PR, and androgen receptor expression [60]. PSA-secreting BC tumors are also typically classified as a lower histologic grade, predicting a better prognosis and outcome [60]. A protease inhibitor, α1-antichymotrypsin (SERPINA3) was also uniquely secreted by MCF-7cells (Fig 4A). Interestingly, PSA and SERPINA3 interact in human serum. In prostate disease, higher circulating levels of PSA-bound SERPINA3 than free PSA are indicative of prostate cancer but not benign hyperplasia [61]. It is intriguing to consider that ERα-positive BC might resemble prostate cancer in the behavior of these two proteins and that a serum profile could similarly indicate BC tumorigenicity. Research is ongoing to produce a PSA blood test that could be predictive of BC in women [62].

### Exosomal proteins

**Fig 5:**
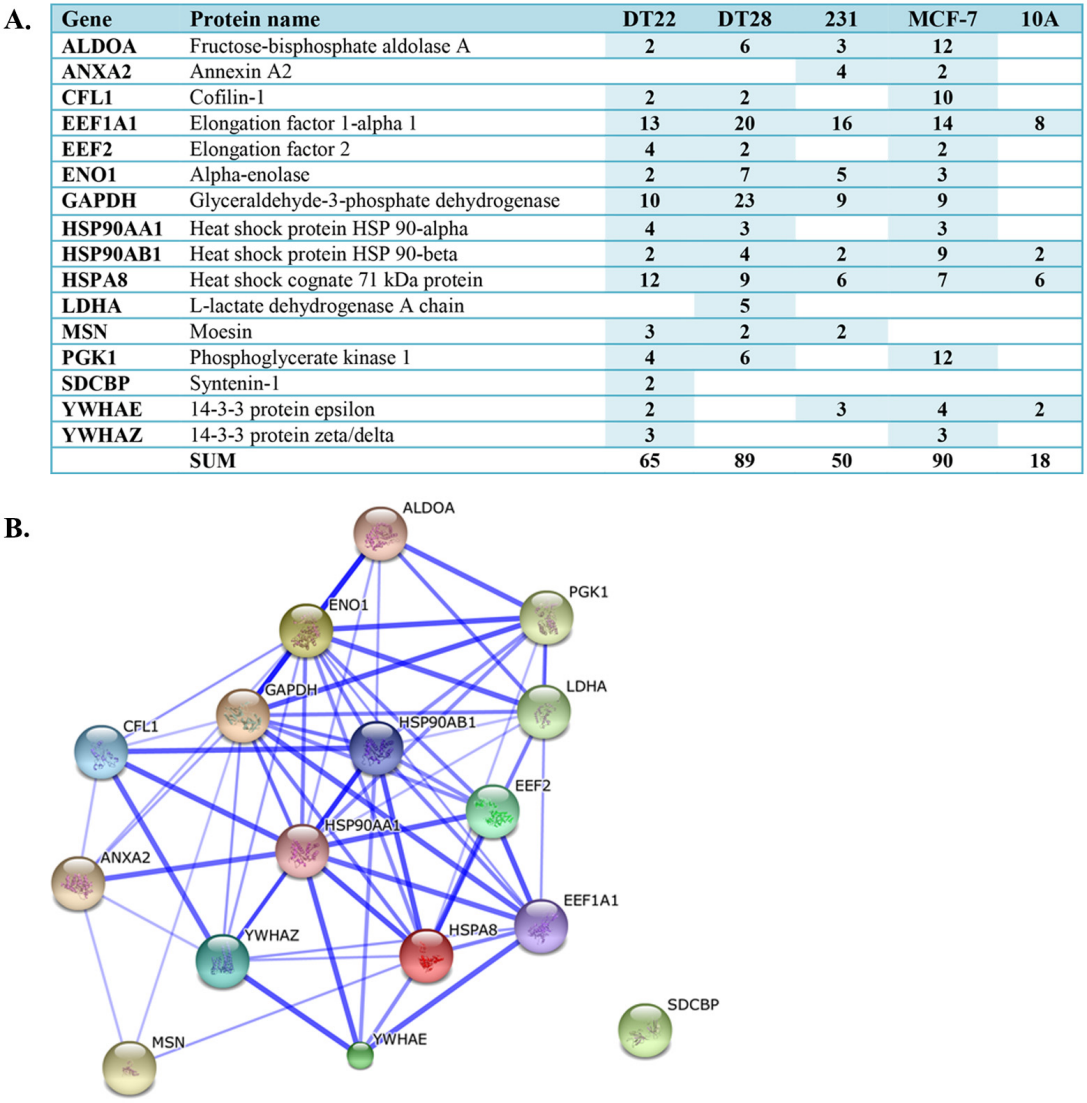
Putative exosomal proteins detected in the CM of BC and MCF-10A cells. A. The number of SCs identified in the CM of each cell line is indicated. MDA-MB-231 and MCF-10A cells are labeled as 231 and 10A, respectively. NSAF data can be found in S3 Table. B. STRING analysis illustrates the numerous complex interactions possible among the putative exosomal proteins. Thicker lines reflect a higher confidence score. (Abbreviations: CM = conditioned medium, BC = breast cancer, SC = spectral counts)

Exosomes are small (40–100 nm) vesicles released by cells throughout the body that contain a variety of molecules including proteins, coding and non-coding RNA, lipids, and metabolites. Exosomes may also play an important role in cancer progression [63], possibly by reprogramming the transcriptome of target cells in a way that fosters tumorigenesis [36]. Conceivably, these exosomes may also prepare distal sites for future metastatic colonization. As such, the cancer cell exosome has become a viable and important new target for diagnosis and therapy. Remarkably, of the 25 proteins most often identified in exosomes [64], 16 were present in the CM of our BC cultures (Fig 5A) indicating that a portion of the secreted proteins may be derived from exosomes. It is noteworthy that the CM from each of the BC cell lines contained far more of these putative exosomal proteins (50–90 SCs) than MCF-10A cell CM (18 SCs). Several glycolytic proteins were represented in this group, as well as proteins involved in signal transduction, cytoskeletal organization, protein folding, and translation. Despite the fact that exosomes are primarily implicated in cell-cell communication, an analysis of the 16 putative exosomal proteins in STRING, a database that examines functional protein interactions [65], illustrates a complex network of interactions that, except for SDCBP, may occur among this group of proteins (Fig 5B).

### Insulin-like growth factor binding proteins

Insulin-like growth factor binding proteins (IGFBPs) bind to IGF-I and IGF-II and lengthen the half-lives of these growth factors. The resulting interactions can influence proliferation, differentiation, and apoptosis depending on the binding protein and the cell context, making it difficult to generalize about the role of IGFBPs in oncogenesis despite many studies in different types of cancer [66]. IGFBPs have generated interest due to their key roles in apoptosis and proliferation and as potential drug targets in cancer and other diseases.

**Fig 6:**
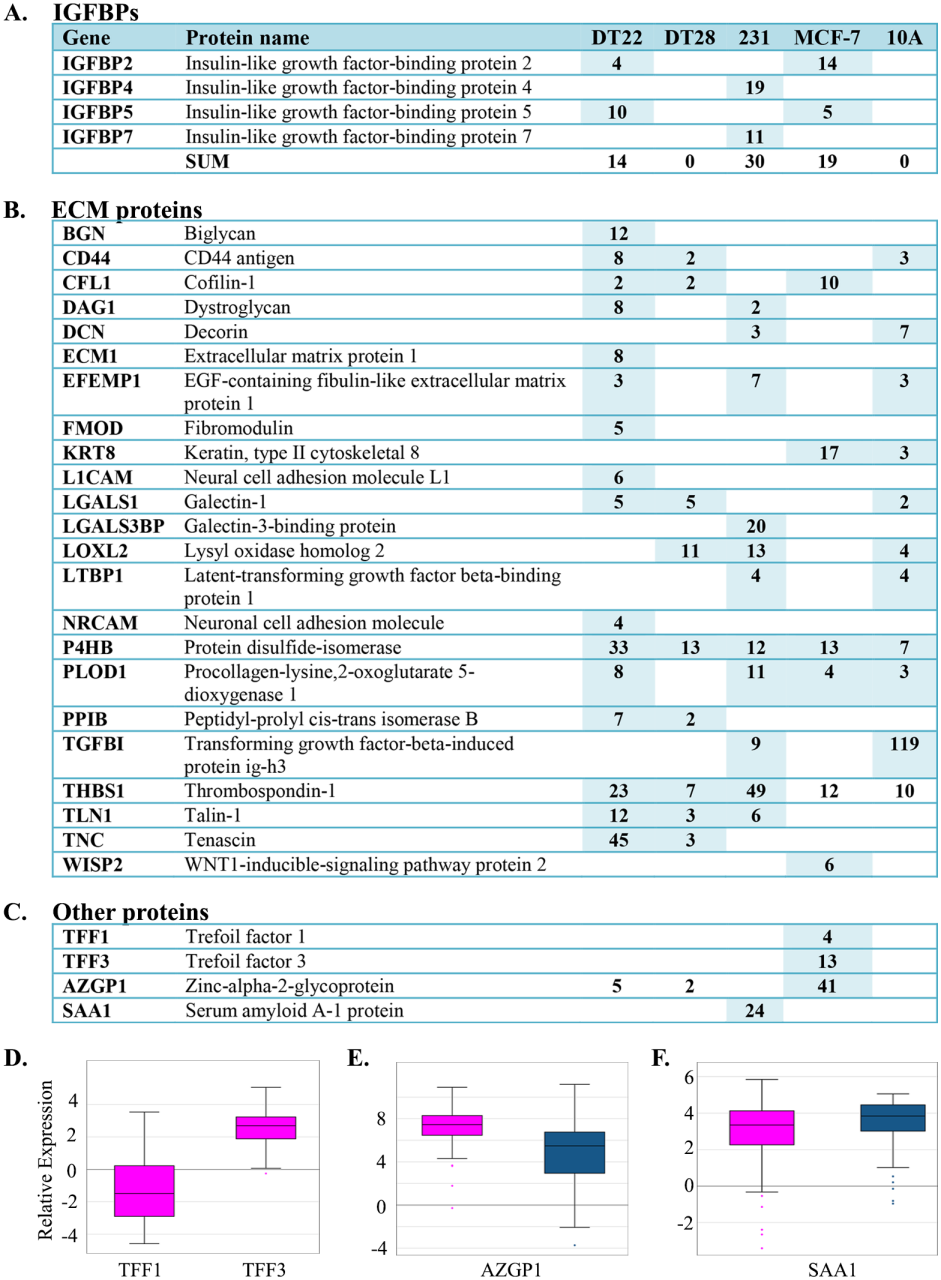
IGFBPs, ECM proteins, and other proteins detected in the CM of BC and MCF-10A cells. A-C. The number of SCs identified in the CM of each cell line is indicated. MDA-MB-231 and MCF-10A cells are labeled as 231 and 10A, respectively. NSAF data can be found in S3 Table. D. BC gene expression data was extracted from TCGA and boxplots were created for luminal A (pink) and TNBC (blue) tumors. Expression values are log2 normalized, tumor matched normal, with normal mammary tissue expression set to 0. (Abbreviations: CM = conditioned medium, BC = breast cancer, SC = spectral counts, TCGA = The Cancer Genome Atlas, TNBC = triple negative breast cancer)

Four of the seven known IGFBPs, IGFBP2, 4, 5, and 7, were detected in the CM with >2 SCs (Fig 6A). Overall, MDA-MB-231 cell CM yielded the highest number of IGFBP SCs and the CM of DT22, MDA-MB-231, and MCF-7 cells each contained two IGFBPs. Interestingly, none of the IGFBPs was detected in either the DT28 or MCF-10A cell CM.

IGFBP4 and IGFBP7 were identified only in MDA-MB-231 cell CM. IGFBP4 has been implicated in the progression of renal cell carcinoma, presumably through activation of Wnt/beta-catenin signaling [67] and is found in the serum of patients with epithelial ovarian cancer [68]. Interestingly, although IGFBP7 has been described as a tumor suppressor in some types of cancer [69,70], a positive correlation between higher IGFBP7 expression and gastric cancer aggressiveness has been observed [71].

Both IGFBP2 and IGFBP5 were detected in the CM of DT22 and MCF-7 cells. IGFBP2 is overexpressed in a wide variety of malignant tumors and this higher expression is reflected in the serum levels of some patients [72]. Most relevant to our study, IGFBP2 is up-regulated in BC but not in benign breast lesions and may be useful as an indicator of malignancy [73]. IGFBP2 has also been detected in the serum of ovarian cancer patients [74] and in the blood of glioma patients with plasma levels correlated to disease-free survival [72]. IGFBP5 has multiple roles in the maintenance of healthy cell function as well as the promotion of metastasis [75]. The expression of IGFBP5 correlates with axillary lymph node invasion in ERα-positive BC and predicts a poor outcome for ERα-negative lymph node positive patients [76].

### ECM and adhesion proteins

MCF-10A cells secreted a remarkable amount (119 SCs) of transforming growth factor-beta-induced protein Ig-H3 (TGFBI, Fig 6B), a protein that plays an important role in cell-matrix interactions. TGFBI is associated with both cancer inhibition [77,78] and progression [77–80]. As the name suggests, this protein is induced by transforming growth factor beta-1, a cytokine that is contextual and multipotent [81]. TGFBI protein affiliates with multiple types of collagen in the ECM and modulates cell adhesion as it interacts with a variety of integrins. Thus, TGFBI may play a role in the highly orchestrated and restricted growth program of MCF-10A cells in 3D culture.

Interestingly, our TNBC cell lines secreted adhesion proteins not typically associated with mammary epithelial cells. DT22 cells uniquely secreted biglycan (BGN), extracellular matrix protein 1 (ECM1), neural cell adhesion molecule L1 (L1CAM), and neuronal cell adhesion molecule (NRCAM) as well as a remarkable amount of tenascin (TNC). BGN plays a role in inflammation and has been implicated in angiogenesis and cell migration [82]. ECM1 is associated with metastasis through the lymphatic system [83]. Serum levels of TNC are elevated in BC patients, with higher grade tumors yielding higher serum levels [84]. Secretion of protein disulfide-isomerase (P4HB) and procollagen-lysine,2-oxoglutarate 5-dioxygenase 1 (PLOD1) was generally higher in the BC cell secretomes than in the MCF-10A secretome. Both of these proteins are implicated in hypoxia-driven remodeling of the ECM leading to increased tissue stiffness and metastatic spread [85]. We previously demonstrated that P4HB is more highly expressed in invasive BC than in ductal carcinoma in situ, benign hyperplasia, or normal mammary epithelial cells [86]. MDA-MB-231 cells uniquely expressed galectin-3 binding protein (LGALS3BP), a protein that modulates cell-cell and cell-matrix interactions. Elevated levels of LGALS3BP have been reported in the serum of BC, lymphoma, pleural mesothelioma, and non-small cell lung cancer patients [87] and are correlated with poor outcomes [88]. LGALS3BP may also play a role in angiogenesis and immune evasion by tumor cells [87]. Thrombospondin-1 (THBS1) is also more highly represented in the CM of MDA-MB-231 cells (49 SCs) and is more moderately represented in the CM of DT22 cells (23 SCs). THBS1, like LGALS3BP, modulates cell-cell and cell-matrix interactions. High expression of THBS1 promotes angiogenesis in liver cancer [89] and THBS1 is over-expressed in esophageal squamous cell carcinoma [90].

More recent discoveries describe development of the pre-metastatic niche, an intriguing process of primary tumors chemically signaling distal sites in the body in preparation for future colonization by metastasized cells. Targeting this process has rich therapeutic potential since it is generally metastasis and not the primary tumor that is ultimately lethal. Lysyl oxidase (LOX), a protein involved in the cross-linking of collagen fibrils, has been implicated in the production of osteolytic lesions, which provide a platform for future TNBC cell colonization [37]. LOXL2, a protein with very similar function to LOX, was secreted in greater amounts by MDA-MB-231 cells (Fig 6B). Hence, the presence of lysyl oxidases in the serum of some TNBC patients might have predictive value for development of malignant bone lesions and could help dictate treatment and monitoring protocols. Interestingly, simtuzumab, a humanized monoclonal antibody, binds to LOXL2 protein and is currently in Phase II clinical trials for treatment of pulmonary fibrosis (clinicaltrials.gov NCT01769196) and is being explored as a therapeutic in colorectal cancer (clinicaltrials.gov NCT01479465).

### Other proteins

MCF-7 cells were unique in secreting trefoil factor 1 (TFF1), widely known as pS2, and trefoil factor 3 (TFF3, Fig 6C). Trefoil factors are a group of three proteins involved in the process of mucosal repair. They are essential rapid responders to mucosal injury and are thus important for maintaining the integrity of tissue lining the intestines and lungs, but the processes that they orchestrate including angiogenesis, motility, anti-apoptosis, and anti-anoikis are similar to the processes that cancer cells exploit to metastasize [91,92]. The pS2 gene is exquisitely estrogen responsive and is used as a universal marker of estrogen responsiveness in MCF-7 cells. Although induced by estrogen and correlated with increased motility of MCF-7 cells in culture [93], higher expression of pS2 has been correlated with decreased aggressiveness [94] and longer recurrence-free survival. Similarly, in normal human bronchial epithelial cells, both TFF2 and TFF3 act as inducers of cell motility in the rapid repair of damaged epithelium [95]. The hijacking of this motility function could possibly promote BC metastasis [96]. It has been suggested that decreased expression of TFF1 and increased expression of TFF3 are associated with a poorer prognosis in gastric cancer. This imbalance is reflected in the TCGA tumor data, in which TFF1 expression is decreased and TFF3 expression is increased in luminal A tumors compared to normal tissue (Fig 6D).

High protein levels of AZGP1 are associated with BC tissue that is less invasive [97]. Thus, the non-metastatic phenotype of MCF-7 cells is reflected in the high level of secretion of AZGP1 by these cells (41 SCs, Fig 6C). Our findings are also in agreement with TCGA BC data for AZGP1, since expression of this protein is higher in the typically less aggressive ERα-expressing tumors (Fig 6E). High expression of AZGP1 in prostate [98,99] and gastric [100] cancer has been linked to delayed recurrence and may be due to AZGP1-mediated down regulation of mTOR signaling and fatty acid synthesis [101].

MDA-MB-231 cells were unique in their secretion of serum amyloid A-1 (Fig 6C, SAA1, 24 SCs). Interestingly, SAA1 is associated with chronic inflammation, is elevated in the plasma of patients with lung and renal cancer, and has been evaluated as a diagnostic and prognostic marker in lung cancer [102]. TCGA BC data indicate that SAA1 expression is increased in both luminal A and TNBC tumors compared to normal mammary tissue with TNBC tumors having a slightly higher average expression (Fig 6F).

### Clinical implications

**Fig 7:**
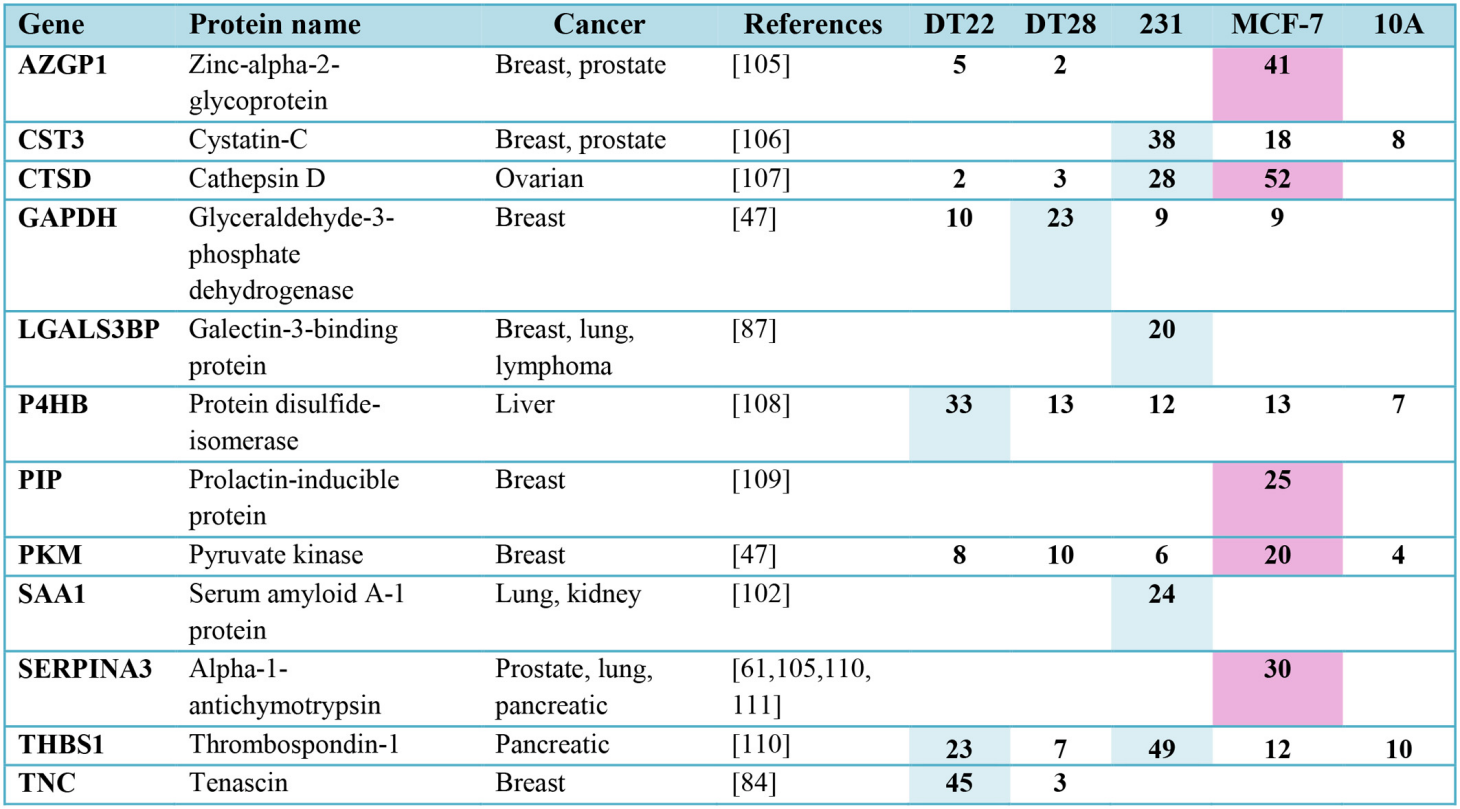
Secreted proteins identified in the CM of BC cell lines (≥ 20 SCs) have been detected in the serum of cancer patients. Higher secretion levels by ERα-positive (pink) and TNBC (blue) cell lines are indicated. NSAF data can be found in S3 Table. (Abbreviations: CM = conditioned medium, BC = breast cancer, SC = spectral counts, TNBC = triple negative breast cancer) [105–111]

It is intriguing to contemplate that a tumor’s secretome, ideally detected through serum analysis, might predict disease aggressiveness as well as define specific strategies for survival and growth that might be clinically targetable. Although the study of the secretomes of BC cell lines has limitations when trying to extrapolate to actual tumor behavior, questions can be addressed that are not possible to study *in vivo*. Using 3D cultures to better model the tumor microenvironment, we demonstrated that many of the secreted proteins we identified play important roles in oncogenic signaling. Intriguingly, several of the MCF-7 and TNBC cell secretion patterns matched gene expression patterns of luminal A and TNBC tumors in the TCGA BC database, thus validating the use of carefully selected cell lines in this type of study. Examination of recurrence free survival data demonstrated the profound impact that many of these secreted proteins have on the survival of BC patients in the clinic. Most importantly, each of the secreted proteins we identified with 20 or more SCs, except for CPE, has been detected in the serum of patients with different types of cancer (Fig 7) and may be useful as markers for BC detection, disease recurrence and progression, or therapeutic intervention. Although CPE has not been detected in serum, it has been implicated as a marker of tumor growth, metastasis, and recurrence in hepatocellular carcinoma [103], pheochromocytomas, and many non-endocrine types of cancer [104]. It may be useful to screen blood for the proteins listed in Fig 7 for BC detection and classification of ERα-responsive (PIP and SERPINA3) and TNBC (LGALS3BP, SAA1, and TNC) tumors.

It was interesting to note that the BC cell lines shared protein signatures and had unique secreted proteins indicating that each tumor evolves its own program for promoting oncogenesis and cancer progression. As indicated in Fig 7, the TNBC cell lines studied here did not group into an easily defined category. Thus, the current simplified clinical classification of TNBC tumors needs to give way to more definitive tumor analyses in order to create more appropriate treatment and monitoring protocols. Indeed, similarities in secretion can arise from tumors originating in different tissues requiring that cancer ultimately be categorized by the pattern of protein expression rather than the tissue of origin. Finally, intriguing observations, such as increased protease secretion correlated with longer recurrence-free survival, need to be addressed if we are to better understand oncogenesis and metastasis. To this end, the synthesis of cell line secretory behavior, TCGA BC data, Kaplan-Meier analysis, and published studies examining serum expression across all types of cancer can yield important clues to identify the most critical and prognostic proteins in the pursuit of accurate diagnosis, targeted treatment, and effective monitoring of disease progression.

## Supporting Information

S1 Table Computer code for extracting BC TCGA data used to create boxplots of gene expression in luminal A and TNBC tumors. (PDF)

S2 Table MS data derived from the CM of BC and MCF-10A cells. The number of SCs and NSAF normalized data for each protein identified in the CM of each cell line is indicated.
(XLSX)

S3 Table SC and NSAF data for Figs 2–7. (XLSX)
