# Supplementary material for: Integration of Breast Cancer Secretomes with Clinical Data Elucidates Potential Serum Markers for Disease Detection, Diagnosis, and Prognosis
Source: PLoS One. 2016 Jun 29;11(6):e0158296. doi: 10.1371/journal.pone.0158296 (PMC4927101; doi:10.1371/journal.pone.0158296)
Supplement: S1 Table — (PDF) [file pone.0158296.s001.pdf]

```ruby
#!/usr/bin/ruby -w

### globals
$tumor_id_key = "Hybridization REF"
$clinical_filename = 'nationwidechildrens.org_clinical_patient_brca.txt'

def main
  gene_ar = get_level3_data
  keys = build_hash_of_level3_keys( gene_ar )
  output_tumor_ids( gene_ar )
  output_level3_data( gene_ar, keys )

  get_clinical_data( gene_ar )
  output_clinical_data( gene_ar )

end



class Gene
  attr_accessor :tumor_id, :hash, :clinical

  def initialize
    @tumor_id = nil
    @hash = nil
    @clinical = nil
  end

end



class Clinical
  attr_accessor :tumor_id, :ar

  @@headers = nil

  def initialize
    @tumor_id = nil
    @ar = nil
  end

  def self.headers
    return @@headers
  end

  def headers=( headers )
    @@headers = headers
  end

end



def get_level3_data

  ### array of hashes, one hash per data file
  ar = Array.new

  ### parse data from *.tcga_level3.data.txt files
  Dir.open( '.' ).each do |d|
    next unless /tcga_level3\.data\.txt$/.match( d )

    g = Gene.new
    h = Hash.new
    File.open( d ).each_line do |line|
      line.chomp!
      key, value = line.split( "\t" )
      h[ key ] = value
    end

    g.tumor_id = h[ $tumor_id_key ][0..11]
```

```
    g.hash = h
    ar << g
  end

  return ar
end



def build_hash_of_level3_keys( ar )
  ### build list of unique keys from data files
  keys = Hash.new
  ar.each do |p|
    p.hash.each do |k,v|
      keys[ k ] = true
    end
  end

  return keys.keys.sort
end



def output_tumor_ids( ar )

  ### output row of all tumor ids
  # first a blank cell, and then the label
  print "\t#{ $tumor_id_key }\t"

  ar.each do |p|
    print "#{ p.hash[ $tumor_id_key ] }\t"
  end
  print "\n"

end



def output_level3_data( ar, keys )

  ### output all other keys and data
  keys.each do |k|
    # label all of these records as genes for easy sorting
    print "gene\t"

    # print label
    print "#{ k }\t"

    ar.each do |p|
      print "#{ p.hash[ k ] }\t"
    end
    print "\n"

  end

end



def get_clinical_data( gene_ar )

  ### parse data from each line of clinical file
  File.open( $clinical_filename ).each_with_index do |line, i|
    line.chomp!
    fields = line.split( "\t" )

    c = Clinical.new
    # headers are in line 0
    c.headers = fields if i == 0

    # skip lines 1 and 2
    next if i < 3
```

```

    c.ar = fields
    c.tumor_id = c.ar[ 1 ]

    # attach clinical data to gene data
    g = gene_ar.find { |x| x.tumor_id == c.tumor_id }
    if g.nil?
      #puts "ERROR: Can't find tumor id in gene data: #{ c.tumor_id }"
      next
    end
    g.clinical = c

  end

end



def output_clinical_data( gene_ar )

  ### output all other keys and data
  Clinical.headers.each_with_index do |header, i|
    # label all of these records as genes for easy sorting
    print "clinical\t"

    # print label
    print "#{ header }\t"

    gene_ar.each do |g|
      unless g.clinical.nil?
        print "#{ g.clinical.ar[ i ] }\t"
      else
        print "\t"
      end
    end
    print "\n"

  end
end

# run application
main()
```
